# COVID-19 y salas de análisis del riesgo en salud pública en cuatro departamentos fronterizos de Colombia

**DOI:** 10.7705/biomedica.6142

**Published:** 2022-03-01

**Authors:** Claudia Marcela Muñoz, Marcela Rocío Arias, Martha Patricia López, Laura Victoria Ortiz, Natalia María Carrillo, Luis Antonio Alvarado, Andrea Morillo

**Affiliations:** 1 Programa de Entrenamiento en Epidemiología de Campo-FETP Colombia, Dirección de Vigilancia y Análisis de Riesgo en Salud Pública, Instituto Nacional de Salud, Bogotá, D.C., Colombia Dirección de Vigilancia y Análisis de Riesgo en Salud Pública Instituto Nacional de Salud Bogotá D.C. Colombia; 2 Dirección de Vigilancia y Análisis de Riesgo en Salud Pública, Instituto Nacional de Salud, Bogotá, D.C., Colombia Dirección de Vigilancia y Análisis de Riesgo en Salud Pública Instituto Nacional de Salud Bogotá D.C Colombia

**Keywords:** infecciones por coronavirus/epidemiología, pandemias, gestión de riesgos, control sanitario de fronteras, Coronavirus infections/epidemiology, pandemics, risk management, border sanitary control

## Abstract

**Introducción.:**

La gestión del riesgo de salud pública en Colombia es liderada por el Instituto Nacional de Salud. Ante la emergencia de la COVID-19, se articularon acciones de respuesta mediante salas de análisis del riesgo y se fortaleció la vigilancia en los puntos de entrada al país.

**Objetivo.:**

Analizar las fases de implementación y mantenimiento de las salas de análisis del riesgo de COVID-19 en cuatro departamentos fronterizos de Colombia.

**Materiales y métodos.:**

Se hizo un estudio cualitativo de salas de análisis del riesgo en salud pública para COVID-19. Se revisaron la documentación y los datos generados en el periodo de marzo a junio de 2020 en Amazonas, Vichada, Guainía y Putumayo, y se hicieron entrevistas semiestructuradas a personas clave, las cuales se analizaron con el aplicativo NVivo plus, versión 11, en tres ciclos: codificación abierta, establecimiento de categorías emergentes y modelación mediante el análisis de las debilidades y fortalezas detectadas.

**Resultados.:**

Se determinaron los componentes de la estructura de comando de incidentes y las relaciones entre las áreas de salud pública. Se encontraron fortalezas en la integración de las áreas, el manejo de la información en tiempo real, la vigilancia en las fronteras y las capacidades de los equipos de respuesta inmediata. Se detectaron debilidades en los procesos de planeación, vigilancia comunitaria y comunicación del riesgo.

**Conclusiones.:**

Las salas de análisis del riesgo constituyen un esfuerzo conjunto del nivel local y nacional que han promovido la participación articulada de los actores, para analizar la información y optimizar la respuesta organizada durante la pandemia de COVID-19.

## Abstract (en)

**Introduction:**

Public health risk management in Colombia is led by the *Instituto Nacional de Salud*. In the face of the COVID-19 emergency, response actions centered on the implementation of risk analysis rooms and the strengthening of surveillance at points of entry into the country.

**Objective:**

To analyze the implementation and maintenance phases of the COVID-19 risk analysis rooms in four border departments of Colombia.

**Materials and methods:**

We conducted a qualitative study of public health risk analysis rooms for COVID-19. We reviewed the documentation and data generated from March to June, 2020, in the departments of Amazonas, Vichada, Guainía, and Putumayo. We did semi-structured interviews with key actors and analyzed the answers using the NVivo plus version 11 application in three cycles: open coding, identification of emerging categories, and modeling by analyzing the identified strengths and weaknesses.

**Results:**

We identified the components of the incident command structure and the relationships between the public health areas. Strengths were evidenced in the integration of the areas: the management of information in real time, the border surveillance and the capabilities of rapid response teams, while weaknesses appeared in planning, community surveillance, and risk communication processes.

**Conclusions:**

Risk analysis rooms constitute a joint effort at the national and local levels which has promoted the articulated participation of all actors in the analysis of information and the optimization of an organized response during the COVID-19 pandemic.

Las emergencias en salud pública incluyen desastres, brotes epidémicos y conflictos en la población. En los últimos años, se han desarrollado programas a nivel global para atender las emergencias en las naciones; es así como, en el 2016, la Organización Mundial en Salud (OMS) estableció el programa de emergencias sanitarias que hoy se implementa en la mayoría de los países ^1^^,^^2^.

Su enfoque en el ciclo de gestión del riesgo en salud permite determinar el grado de vulnerabilidad y amenaza, analizar las posibles consecuencias en términos de impacto y efectos, incorporar acciones de prevención y preparación previas a la emergencia, y generar respuestas oportunas de recuperación una vez atendida la situación, lo que facilita la toma de decisiones y la comunicación del riesgo ^2^^,^^3^. Mediante este mecanismo, se establecen procesos para reducir la aparición de enfermedades a niveles aceptables mediante medidas de prevención y modificar los factores de riesgo, con el fin de evitar la enfermedad y mitigar sus efectos ^4^.

En el Plan Decenal de Salud Pública 2012-2021, se establecieron líneas operativas para la gestión del riesgo mediante un conjunto de acciones ejecutables en la población ^5^. En el 2016, el Instituto Nacional de Salud fortaleció las acciones de gestión del riesgo, consolidando un área enfocada en la detección de emergencias y brotes, y la reacción inmediata; además, estandarizando los procedimientos y actividades del sistema de alerta temprana, la sala de análisis del riesgo, el centro de operaciones de emergencias, los equipos de reacción inmediata y la comunicación del riesgo en salud pública ^6^. La sala de análisis del riesgo garantiza la articulación entre sectores y dentro de ellos, ante situaciones inesperadas que afecten las condiciones de vida de las poblaciones y de su condición de salud ^7^.

El 11 de marzo de 2020, la OMS declaró la pandemia de COVID-19 y emitió recomendaciones para activar los mecanismos de reacción ante la emergencia en los diferentes niveles de los países ^8^. La rápida propagación de la pandemia a nivel mundial activó los protocolos para detener su diseminación. En Colombia, se confirmó el primer caso el 6 de marzo del 2020 ^8^ y se declaró la emergencia sanitaria el 12 de marzo del 2020 ^9^. Antes de que se reportaran casos en el país, el Instituto Nacional de Salud había implementado las salas de análisis del riesgo en diferentes departamentos y distritos mediante gestores, para analizar la información en tiempo real y tomar decisiones frente a los casos en los municipios según las directrices establecidas ^10^.

Ante las amenazas detectadas, se estableció la sala de análisis del riesgo para medir el riesgo y coordinar las acciones según lo establecido conjuntamente con el Grupo de Gestión de Riesgo en Salud Pública del Instituto Nacional de Salud y la norma técnica ICONTEC ISO 31000 ^11^. A partir del diagnóstico inicial, se dieron orientaciones sobre las acciones de preparación y respuesta, la capacidad de los laboratorios de salud pública, la gestión en salud pública y la comunicación del riesgo ^12^.

Entre las acciones de vigilancia de la COVID-19 en Colombia, se enfatizaron: el fortalecimiento del control en los puntos de entrada a los territorios, como aeropuertos y terminales marítimos y terrestres; la gestión de la notificación de los eventos al Sistema de Vigilancia en Salud Pública (Sivigila); la verificación y el seguimiento de rumores y de la información en medios de comunicación relacionados con la COVID-19; el seguimiento de casos y el rastreo de contactos; la investigación epidemiológica de campo y la generación de reportes de situación, y el entrenamiento de los equipos de respuesta inmediata ^13^. Asimismo, se determinaron los beneficios potenciales del análisis de la implementación y mantenimiento de las salas de análisis del riesgo para establecer cómo el proceso se adaptaba a las necesidades de respuesta en las entidades territoriales y se integraba a los procesos rutinarios de la vigilancia en salud pública. Ello permitió realimentar a las partes interesadas en los beneficios de la sostenibilidad del proceso en el tiempo, y en la necesidad de fortalecer la actividad con recurso humano capacitado, lecciones aprendidas, y elementos de ofimática para mejorar el procesamiento, análisis y divulgación de la información de brotes y emergencias.

Un beneficio adicional es la gestión de las emergencias en salud pública a nivel territorial, la cual facilita el análisis de la información con la participación de las diferentes partes involucradas para la adopción de las mejores decisiones. En ese marco, el objetivo del presente estudio fue analizar las fases de implementación y mantenimiento de la sala de análisis del riesgo y la gestión de la respuesta a la pandemia de COVID-19 en cuatro departamentos fronterizos de Colombia.

## Materiales y métodos

Se hizo un estudio cualitativo y descriptivo que contempló dos tipos de análisis, el documental ^14^ y el de carácter fenomenológico ^15^, en la secretarías de salud de cuatro departamentos: Amazonas, Putumayo, Guainía y Vichada.

El análisis documental incluyó la revisión y consolidación de los documentos generados en los procesos de instalación y mantenimiento de las salas de análisis del riesgo en los departamentos entre marzo y junio del 2020. Se clasificó la información según los criterios establecidos por el Grupo de Gestión de Riesgo del Instituto Nacional de Salud en respuesta al objetivo de cada criterio (cuadro 1). Una vez clasificados los documentos, se recopiló y se comparó la información por departamento, buscando párrafos de interacción y similitud, así como aquellos con información exclusiva de cada entidad, para luego analizar los hallazgos y organizarlos para su visualización ^15^.

**Cuadro 1:** Criterios de análisis de la instalación y mantenimiento de las salas de análisis del riesgo para la COVID-19 en los departamentos de Amazonas, Guainía, Vichada y Putumayo, marzo a junio de 2020

Criterio	Objetivo	Actividades	Documentos verifcados
1. Gestión estratégica	Evaluación y seguimiento	Diagnóstico de capacidad de respuesta: conformación de comités de respuesta, disponibilidad de talento humano, insumos, plan de acción y medios para COVID-19 y determinación de nivel de riesgo	Informe de diagnóstico inicial de capacidad de respuesta de la entidad y matriz de riesgo analizada
		Reunión semanal de seguimiento de actividades del plan de acción de la SAR COVID-19 Organización del personal de los equipos de respuesta inmediata (ERI) para apoyar las acciones de contingencia ante la COVID-19	Actas de instalación y mantenimiento semanal de la SAR para la COVID-19 Acta con la distribución de actividades y responsables de la SAR y conformación de los ERI
			Actas de entrenamientos en uso de elementos de protección personal y procedimientos de vigilancia
2. Laboratorio de salud pública	Determinación de capacidad diagnóstica	Diagnóstico, actualización y gestión de insumos y personal	Informes de gestión de insumos y personal
		Actualización de la red de laboratorios, colaboradores y estado de las muestra	Documento de actualización de la red de laboratorios, colaboradores y seguimiento de muestras tomadas
		Actualización de las directrices para la vigilancia por laboratorio de virus respiratorios: insumos para muestras proyectadas, contratación de personal y entrenamiento continuo para toma de muestras	Actas de instalación y mantenimiento semanal de la SAR para COVID-19
3. Sanidad y vigilancia	Vigilancia puntos de entrada	Articulación de la SAR para COVID-19 de la entidad territorial y sanidad portuaria, generación de estrategias de vigilancia en puntos de entrada (aeropuertos, puertos marítimos, puertos en ríos, pasos fronterizos)	Actas de instalación y mantenimiento semanal de la SAR para la COVID-19
4. Vigilancia en salud pública	Determinación de capacidad de gestión y respuesta	Seguimiento y monitoreo semanal de la situación epidemiológica y la notifcación de la morbilidad por infección respiratoria aguda e identifcación de conglomerados o comportamientos inusuales en la notifcación de los casos	Informe ejecutivo de reporte de situación (SITREP) diario y semanal
		Detección y verifcación de rumores y monitoreo de medios de comunicación	SITREP diario y semanal, matrices de seguimiento de rumores
		Gestión de los casos confrmados: historias clínicas, investigaciones epidemiológicas de campos, matriz de seguimiento a contactos, fcha de notifcación y resultado	IEC, matriz de seguimiento a contactos, fcha de notifcación y resultado de laboratorio
		Análisis de vigilancia en salud pública de la COVID-19 diario por cada entidad	Infografías, tablero de mandos, bolet epidemiológicos y otros similares
5. Centro regulador de urgencias y emergencias/ Prestación de servicios	Regulación de los procesos de atención	Articulación de vigilancia con las áreas de prestación de servicios y aseguramiento para fortalecer la capacidad instalada y la red de prestación de servicios: urgencias, hospitalización y unidad de cuidados intensivos, adecuación de procesos de referencia y contrarreferencia, ocupación de los servicios	Actas de instalación y mantenimiento semanal de la SAR para la COVID-19
6. Comunicación del riesgo	Comunicación e información a la comunidad	Seguimiento del plan de medios, identifcación de voceros ofciales y omunicaciones emitidas	Actas de instalación y mantenimiento semanal de la SAR para la COVID-19

Fuente: informes de diagnóstico e implementación de las SAR (Amazonas, Guainía, Vichada y Putumayo)

Fuente: SAR - Instituto Nacional de Salud, revisión documental

El análisis fenomenológico giró en torno a la comprensión de la experiencia vivida, siguiendo el método de Martin Heidegger ^16^ a partir de una entrevista semiestructurada ^17^. Las preguntas de la entrevista buscaban entender el impacto de las salas de análisis del riesgo en la pandemia directamente con quienes las lideraron y establecieron en sus territorios. Mediante un muestreo intencionado, se seleccionaron cuatro profesionales de cada secretaría departamental que cumplieran con los criterios de inclusión: participación en la respuesta a la pandemia, tener un cargo de liderazgo y experiencia laboral de más de dos años en salud pública. En cuanto a los ítems de la entrevista, se indagó sobre la respuesta a la pandemia y el establecimiento de las salas de análisis del riesgo, la integración con diferentes áreas para la toma de decisiones, el foco de los esfuerzos para establecer acciones de respuesta y las actividades del plan de acción, que permitieron mejorar los procesos de contención de la emergencia y la detección de los principales problemas y desafíos que se presentaron.

Antes de la entrevista, se hizo una presentación verbal y se entregó en físico el documento con el objetivo de la investigación, y se solicitó la participación voluntaria y la aprobación de los seleccionados para grabarlos mediante la firma del consentimiento informado. Para el análisis, se siguió la propuesta de Taylor-Bogda, que asegura el rigor metodológico y un mayor respaldo en la interpretación de los resultados. Por ello, las entrevistas se grabaron y se transcribieron textualmente para dar credibilidad al análisis de los datos ^18^.

Todos los participantes hicieron la lectura reflexiva del material discursivo transcrito, para determinar las ideas principales y luego analizarlas utilizando el aplicativo NVivo plus, versión 11. Se hizo un ciclo de codificación, uno de detección de categorías emergentes, así como la codificación axial de los textos significantes, y se determinaron las relaciones entre las diferentes categorías. En cuanto a la saturación, se estableció cuando los nuevos datos no aportaron más información en las categorías que emergieron. Por último, se hizo la modelación mediante el análisis de las debilidades y fortalezas detectadas, para transferir los hallazgos a los resultados y a la discusión sobre el análisis llevado a cabo ^19^. Se garantizaron los criterios de rigor metodológico del enfoque cualitativo, estableciendo: la credibilidad de la información (la veracidad de los documentos y las entrevistas transcritas); su transferibilidad (recolección y descripción de la información a partir de los documentos de la sala de análisis del riesgo y selección de los participantes con base en criterios); la dependencia (réplica paso a paso), y la posibilidad de confirmación (consenso entre los investigadores y las citas extraídas de las entrevistas realizadas).

### Consideraciones éticas

El estudio no entrañaba riesgo alguno según la Resolución 08430 de 1993 ^20^. Se emplearon técnicas y métodos de investigación documental retrospectivos, y no se hizo ninguna intervención o modificación intencionada de las variables biológicas, fisiológicas, psicológicas o sociales de los participantes, los cuales participaron de forma voluntaria y firmaron un consentimiento informado después de que les explicaron los objetivos del estudio y la finalidad de la entrevista. Además, se garantizó la privacidad de los informantes y la confidencialidad de la información mediante la asignación de códigos alfanuméricos.

## Resultados

### Área de estudio

**Figura 1:**
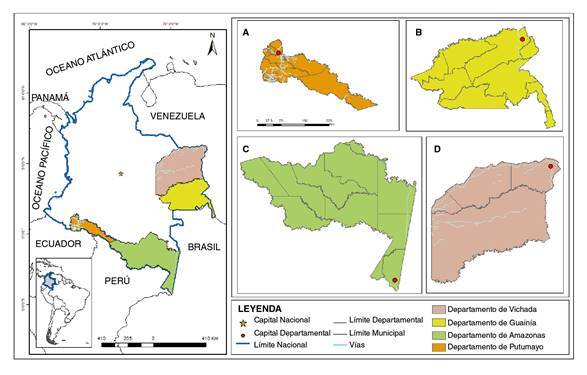
Ubicación de los departamentos de Amazonas, Guainía, Vichada y Putumayo.

Colombia se localiza en el noroeste de Suramérica. Limita por vía terrestre con Venezuela, Brasil, Perú, Ecuador y Panamá ^21^. Los departamentos objeto de estudio fueron Amazonas, Guainía, Putumayo y Vichada. El Amazonas limita con Brasil y Perú; Guainía, con Brasil y Venezuela; Vichada, con Venezuela, y Putumayo, con Perú y Ecuador (figura 1). En los cuatro departamentos se implementó la sala de análisis del riesgo de COVID-19 en marzo del 2020, la cual se mantuvo durante la pandemia.

Estos departamentos se cuentan entre los de menor densidad poblacional del país ^22^. Un alto porcentaje de la población en ellos es indígena: 64,9 % en Guainía; 44,3 % en Vichada; 43,4 % en Amazonas y 20,9 % en Putumayo ^23^. Los cuatro hacen parte de la región amazónica colombiana, se ubican principalmente en la selva y no cuentan con vías de acceso a las zonas rurales donde están establecidas las comunidades indígenas ^24^. Amazonas y Guainía están conectados con el resto del territorio nacional únicamente por vía aérea, en tanto que Putumayo y Vichada cuentan con vías terrestres y fluviales, siendo de mayor importancia estas últimas en el departamento de Vichada ^25^. Por su localización, los cuatro departamentos tienen un gran intercambio comercial en la frontera con Brasil, Venezuela, Perú y Ecuador, en especial Amazonas, con un gran movimiento de población entre las fronteras por la importante demanda turística de extranjeros y nacionales ^26^.

### Implementación de la sala de análisis del riesgo de COVID-19

Los cuatro departamentos iniciaron su proceso de instalación de la sala de análisis del riesgo de COVID-19 en marzo del 2020, en el marco de los procesos iniciales de respuesta organizada en cada entidad territorial (cuadro 2). A partir del diagnóstico inicial de cada una de ellas, se establecieron los pasos para la instalación de las salas, y los departamentos se organizaron siguiendo el modelo de comando de incidente y estableciendo las estructuras de gestión de las salas.

**Cuadro 2:** Descripción del diagnóstico de la capacidad de respuesta inicial ante la Covid-19 en los departamentos de Amazonas, Guainía, Vichada y Putumayo

Criterio	DEPARTAMENTO		
	Amazonas	Guainía	Vichada	Putumayo
Estructura inicial de respuesta	Sala de Comando Unifcado Departamental, frecuencia de reunión, diaria	Consejo Municipal de Gestión de Riesgo Departamental, frecuencia de reunión, una vez a la semana	SAR liderada por secretaría departamental de salud, frecuencia de reunión, diaria	Comité de Emergencias y Desastres de la Secretaría de Salud Departamental
Nivel de riesgo y respuesta	Se determinó para los cuatro departamentos un riesgo de nivel IV, es decir, se require una respuesta a todos los niveles: nacional, intersectorial e internacional			
Acciones	Enfocadas a vigilancia en puertos, aeropuertos y pasos fronterizos. Dos reuniones realizadas con autoridades de Brasil y Perú para ﬂujo de información, y cierre y control de fronteras. Se activa el Comité Departamental de Sanidad Portuaria del Amazonas el 03/02/2020 y se implementan acciones de tamización en puntos de entrada al departamento	Acciones enfocadas a centinelas en puntos de entrada con tamizaje a viajeros activados a partir del 03/03/2020. Puntos centinelas localizados en cabecera municipal de capital: puertos ﬂuviales (El Paujil, puerto muelle de carga, balsa de la policía y puerto minero) y Aeropuerto César Gaviria Trujillo	Plan de contingencia enfocado a rutas de atención y detección de casos en aeropuerto, puerto ﬂuvial, comunidad y atención en instituciones prestadoras de servicio de salud (IPS).	Seguimiento de vigilancia epidemiológica rutinaria de infección respiratoria aguda, seguimiento de rumores, capacitación grupos funcionales, entidades administradoras de planes de benefcios de salud (EAPB), entidades del sector público y privado en aspectos generales de la Covid-19. Activación de centros de operaciones de emergencia (COE). Asistencias técnicas a municipios en protocolos de la Covid-19
Recurso humano e insumos	Personal de planta: coordinadora, profesionales de enfermería y una técnica	Personal de planta: dos profesionales de enfermería, dos auxiliares administrativos, un técnico operativo y un auxiliar de enfermería	Dos epidemiólogos en área de vigilancia en salud pública, un técnico de Sivigila y tres apoyos en cada municipio para enlace con secretaría de salud departamental	Ocho profesionales de salud en área de vigilancia en salud pública Tres especialistas en epidemiología
	ERI: 20 ERI, 57 personas: especialistas en epidemiología (5), médicos (2), profesionales (16), técnicos (23) y auxiliares de salud (11), capacitados en directrices para la Covid-19 Laboratorio de salud pública (LSP): personal capacitado para la toma, recepción y envió de muestras	ERI: Coordinadores de cada una de las áreas de secretaría de salud departamental. Disponibilidad por semana de una persona para atención de brotes	ERI integrado por coordinadores de áreas de la secretaría de salud con disponibilidad de dos personas por semana para seguimiento de alarmas	ERI: conformado por médico, epidemiólogo, enfermera de programa y bacteriólogo
	Cuenta con equipo para el análisis molecular (PCR) de las muestras sin certifcar Departamento no cuenta con laboratorios autorizados para realizar PCR para la Covid-19	LSP: personal capacitado para la toma, recepción y envió de muestras. Solo una bacterióloga para toma de muestra Departamento no cuenta con laboratorios autorizados para realizar PCR para la Covid-19	LSP: departamento no cuenta con laboratorios autorizados para realizar PCR para Covid-19.	LSP: personal capacitado para la toma, recepción y envió de muestras Departamento no cuenta con laboratorios autorizados para realizar PCR para la Covid-19
Comunicación	Boletín epidemiológico con actualización diaria, folleto con información básica para la población en general, Cuenta con plan de medios	Ruta de detección y atención para un posible evento de Covid-19 que opera como cadena de llamadas. No cuenta con plan de medios		Educación y comunicación de los aspectos de la Covid-19
Articulación de la población étnica	Reuniones con asociaciones de autoridades indígenas para autorización de socialización de lineamientos divulgación de directrices de prevención de la Covid-19	nformación generada es traducida a cada una de las lenguas de comunidades indígenas presentes en departamento		

Fuente: Informes de diagnóstico e implementación de salas de análisis del riesgo (SAR) (Amazonas, Guainía, Vichada y Putumayo)

### Acciones de mantenimiento de la sala de análisis del riesgo de COVID-19

*Gestión estratégica*. La estructura de los planes de contingencia involucró la organización de los equipos de respuesta inmediata y oportuna, aumentando la capacidad del personal y su disponibilidad semanal. Dichos equipos recibieron capacitación en las directrices y el uso adecuado de los elementos de protección personal, los procesos de información, educación y comunicación (IEC), y los de toma y embalaje de muestras. En cada departamento, se conformaron 20 equipos de respuesta inmediata, cada uno con personal interdisciplinario para la toma de muestras, la investigación de conglomerados y las alertas.

*Laboratorio de salud pública*. Una vez instalada la sala de análisis del riesgo, los departamentos establecieron en su plan de contingencia la capacitación del personal de la red de laboratorios y las instituciones prestadoras de servicios de salud (IPS) para la toma de muestras y su embalaje. Todo el personal recibió medios de transporte viral para hacer el tamizaje de la población vulnerable y de riesgo (Amazonas: 5.200; Guainía: 400; Vichada: 5.500 y Putumayo: 200 medios de transporte viral) durante este periodo, asegurando así la capacidad instalada de cada departamento.

Los departamentos no contaban con laboratorios autorizados para hacer las pruebas de PCR para COVID-19, por lo que se establecieron estrategias de envío de las muestras a los laboratorios certificados. Los envíos se hicieron según la disponibilidad de vuelos de carga o de la Fuerza Aérea Colombiana, así como por medio de la Asociación Nacional de Departamentos. Durante el periodo analizado, Amazonas envío 8.910 muestras; Guainía, 1.051; Vichada, 771, y Putumayo, 730. Se mejoró la capacidad instalada de los laboratorios para la toma y envío de muestras, una de las actividades que mayor coordinación exige para obtener oportunamente los resultados. Todas las entidades analizadas lograron coordinar formas de transporte para agilizar el procesamiento de las muestras; en Guainía, Vichada y Amazonas, el envío aéreo fue la única opción ante la ausencia de vías terrestres de comunicación con laboratorios de referencia.

*Sanidad portuaria*. En los puntos de entrada de frontera y los puertos fluviales y aéreos, se establecieron grupos para el registro de las personas que ingresaban a los departamentos, siguiendo las indicaciones establecidas para el tamizaje de viajeros ^26^, y mediante acciones de sensibilización a la población sobre los síntomas, formas de transmisión y prevención de la COVID-19. A las personas registradas se les tomó la temperatura y se les hizo la tamización, y firmaron actas de compromiso de cumplimiento del aislamiento preventivo.

En Amazonas, se ubicaron los casos entre quienes cruzaban ilegalmente la frontera, en coordinación con Migración Colombia, la Policía Nacional, y las secretarías de gobierno y turismo municipal y departamental, estableciendo el punto principal en la frontera entre Tabatinga y Leticia. En el departamento de Vichada, se establecieron grupos en los puestos de control terrestre, fluvial y aeroportuario de los municipios conjuntamente con los grupos de sanidad portuaria departamental y las secretarías municipales de desarrollo social de Santa Rosalía, Cumaribo, La Primavera y Puerto Carreño. En Guainía, se conformaron nueve grupos extramurales localizados en la frontera nacional (cinco) y en la internacional (cuatro), articulando la vigilancia con la guardia indígena, los capitanes de las comunidades indígenas, los líderes y corregidores, la fuerza pública y los líderes de salud de los municipios limítrofes con Venezuela y Brasil. En Putumayo, se conformaron grupos de vigilancia y tamizaje conformados por auxiliares de enfermería, enfermeros y médicos, en los municipios de Puerto Leguízamo, Puerto Asís, Valle del Guamuez y San Miguel.

Asimismo, los cuatro departamentos continuaron con sus acciones rutinarias de inspección, vigilancia y control de mercancías y alimentos, haciendo énfasis en el cumplimiento de los protocolos de bioseguridad.

*Vigilancia en salud pública*. Los primeros casos positivos de COVID-19 se notificaron en abril en el departamento de Amazonas y, en mayo, en Guainía, Vichada y Putumayo. Hasta el 30 de junio de 2020, se notificaron 2.300 casos en Amazonas, 14 en Guainía, 18 en Putumayo y uno en Vichada.

En todos los departamentos, se hizo el seguimiento de rumores y alertas en la comunidad, así como investigaciones epidemiológicas de campo presenciales y telefónicas en respuesta a las alertas y los casos confirmados; se tomaron las muestras según la definición operativa de caso y se hizo seguimiento de los contactos estrechos de los casos confirmados. En las respectivas salas, se hizo el análisis diario de los datos recolectados mediante búsqueda activa comunitaria e institucional y el análisis de la notificación al Sivigila de los casos de infección respiratoria aguda.

Actualmente, la sala de análisis del riesgo en Amazonas cuenta con talento humano para hacer el seguimiento a los pacientes hospitalizados, el análisis de la mortalidad, y el seguimiento de los casos en las comunidades indígenas y las zonas no municipalizadas.

En Inírida (Guainía), se implementó la ruta de detección y atención de posibles casos de COVID-19, utilizando la cadena de llamadas. La ruta fue difundida en todas las instituciones del municipio, se le entregó a la población para su conocimiento y se hizo búsqueda efectiva en los servicios de salud de casos de sintomatología sugestiva de COVID-19.

El plan departamental de contingencia del Vichada contempló la implementación de rutas de detección y atención para casos probables de COVID-19 en el aeropuerto, el puerto fluvial, el terrestre, la comunidad y las IPS. Dichas rutas fueron divulgadas para incentivar a la población a acudir a los servicios de salud ante cualquier sintomatología sugestiva de COVID-19.

En cuanto a la búsqueda comunitaria activa, se fortalecieron los procesos en los territorios para identificar a las personas sintomáticas y a la población de riesgo en áreas priorizadas, y se fortalecieron los procesos de notificación inmediata al Sivigila de los casos probables y los confirmados. Asimismo, se había hecho un seguimiento focalizado de las alertas epidemiológicas, así como de los conglomerados en diferentes instituciones: batallones, población privada de la libertad y hogares geriátricos.

Se llevaron a cabo acciones de vigilancia en comunidades indígenas mediante el trabajo concertado con los líderes indígenas, acciones de capacitación para fortalecer la vigilancia activa y detectar los casos, y otras para la contención oportuna. Los cuatro departamentos adoptaron y adaptaron las directices nacionales de vigilancia innovadora como parte de las acciones locales ^13^, incluido el tamizaje poblacional, las búsquedas activas comunitarias y la vigilancia digital sindrómica.

*Centro regulador de urgencias y emergencias y prestación de servicios*. Se hicieron capacitaciones sobre los protocolos relacionados con la COVID-19 y los de bioseguridad, así como la actualización continua de las directrices de desinfección y limpieza de ambulancias y de los espacios de atención hospitalaria en empresas de prestación de servicios de salud e IPS, y su seguimiento.

*Comunicación del riesgo*. Se formularon y documentaron los planes de medios para COVID-19 y, en todos los departamentos, se establecieron esquemas informativos por medio de boletines diarios publicados en la página web oficial de cada departamento e infografías. Se hizo el seguimiento a rumores, se publicaron las estrategias de información por medio de los diferentes medios de comunicación, y de educación en salud y bioseguridad dirigidas por el personal sanitario.

### Entrevistas a los participantes

En las entrevistas, se destacaron los textos que representaban las actividades a cargo de las entidades territoriales, aquellas fundamentales para dar respuesta integral a los riesgos y amenazas tras la revisión en las salas de análisis de riesgo, las cuales se plasmaron utilizando una nube de palabras en la que resaltaron términos como “COVID”, “respuesta,” “salud”, “vigilancia,” “pandemia”, “áreas”, “implementación”. En el análisis del discurso de las entrevistas, se establecieron cuatro categorías emergentes: actores de respuesta, salas de análisis del riesgo, problemas y desafíos, y comunicación del riesgo.

### Actores de respuesta

En la categoría “actores de respuesta” se manifestó la experiencia en la articulación de varias áreas para dar respuesta a la pandemia: el laboratorio, el área de prestación de servicios, la vigilancia y los centros reguladores de urgencias y emergencias (CRUE). En esta categoría, se establecieron las acciones de capacitación dirigidas al personal de salud a nivel asistencial y a los operadores de vigilancia en salud pública. Con respecto al alistamiento y las rutas establecidas para lograr una respuesta inmediata, se evidenció el trabajo conjunto con otras áreas y la integración necesaria para atender las situaciones de la pandemia en cada territorio.

*[...] desde que se implements la Salas de Análisis del Riesgo se llegó un poco más a las áreas de prestación de servicio, y CRUE (ACT1001) (sic).*

*[...] una de las cosas que a raíz de la pandemia se han incrementado y se ha fortalecido, son los temas de capacitaciones a todo el personal médico y a los municipios con ayuda ya pues obviamente del área de CRUE y desde el área de prestación de servicios, con médicos ya integrados pues más en el tema obviamente con la integración del personal médico de vigilancia (ACT2002).*

*[...] ya estábamos preparados, ya teníamos establecidas rutas y la forma de cómo actuar en caso que se nos presentara entonces si fue muy favorable para nosotros como departamento (ACT1003) (sic).*

### Sala de análisis del riesgo

En esta categoría, se evidenciaron la estructura organizacional establecida, la integración con otras áreas y la importancia de la toma de decisiones. En cuanto a las fortalezas en la implementación de las salas de análisis del riesgo en los departamentos, los participantes mencionaron la organización de las áreas para hacer analizar la situación y dar respuesta oportuna de manera integral.

*La Sala de Análisis del Riesgo tiene una estructura que nos permite ser más organizados, y analizar conjuntamente la situación y plantear respuestas. Aunque, queriendo que se integren el total de las áreas involucradas en la respuesta, finalizaron varios procesos (ACT1001) [...] (sic).*

*La implementación de la Salas de Análisis del Riesgo obviamente nos ayuda y nos sigue ayudando en este momento para poder tomar decisiones de manera más oportuna y dar respuesta inmediata a las situaciones que se nos presentan (ACT2002).*

*[...] aunque siempre hemos tenido comunicación entre áreas no contábamos con el espacio donde todos estuviéramos a la vez e interactuar para generar acciones que fortalecieran cada una de las áreas como se hizo desde que se implementó la Salas de Análisis del Riesgo (ACT1003) [...] (sic).*

*[...] ha permitido generar un espacio muy valioso donde está conformada por diferentes componentes de prestación de servicios, sanidad portuaria, laboratorio, salud pública y vigilancia, las cuales están unidas y encaminadas al fortalecimiento de las acciones de mantenimiento y seguimiento de respuesta para la COVID-19 (ACT1004) [...] (sic).*

Por otro lado, se destacó la importancia del análisis de la información, y de la participación y el trabajo articulado de diversos agentes, lo que permitió cambiar y fortalecer las acciones de vigilancia en salud pública. Una acción fundamental evidenciada por los participantes fue el fortalecimiento de la capacidad de respuesta y las acciones para mantenerla en los territorios mediante el sistema de gestión del riesgo para detectar alertas tempranas.

*Se convirtió un espacio para un análisis y toma de decisiones con la participación de los diferentes actores de la secretaria de salud y de otras instituciones cuando se requiere y la puesta en común de la situación para que cada uno aporte a las soluciones, y eso no lo estábamos haciendo antes, entonces me parece que es un espacio que se ha ganado (ACT1004) (sic).*

*Creamos unas capacidades de respuesta, que hasta la fecha se mantienen y nos hacen más proactivos y estar dos pasos adelante de cómo debemos reaccionar (ACT1001) (sic).*

### Problemas y desafíos

En esta categoría, se identificaron situaciones relacionadas con las realidades de cada territorio, como la resistencia frente al trabajo articulado, la dificultad para la planeación de procesos, los mecanismos de inducción del personal, los insumos de protección personal y la importancia de la operación del sistema de vigilancia comunitaria.

*Hablando con la verdad el trabajo en equipo, porque cada uno está dispuesto solo a trabajar en su parte y vigilancia y la respuesta ante la pandemia ha sido trabajo en equipo escuchar que dice el laboratorio y que limitantes tiene, escuchar que limitantes tiene vigilancia, como apoya sanidad portuaria, como apoya prestación eso ha sido el mayor desafío que hemos tenido (ACT1004).*

*Al ser tan pocas personas en la institución; siempre vamos a ser los mismos haciendo lo mismo; entonces el hecho de tener la rutina de reunirnos cada semana entonces el que fueran puntuales nos llevó mucho trabajo y pues sentirse obligados de venir a la Sala de Análisis del Riesgo al inicio fue un poco complejo (ACT1003) lo único que no tuvimos o sea en su oportuno momento fue elementos de protección personal, de manera inmediata. Pero yo digo que en el trascurso de 15 días ya fueron llegando (ACT2002) (sic).*

*Llevar la vigilancia a los niveles comunitarios, si nosotros tuviéramos un sistema de vigilancia comunitaria más fuerte el trabajo fuere un poco más fácil. Si tuviéramos el empoderamiento de la comunidad. Si tuviéramos el empoderamiento de la comunidad. Si las Juntas de Acción Comunal reportaran, si el promotor reportara (ACT1001) (sic).*

### Comunicación del riesgo

**Figura 2:**
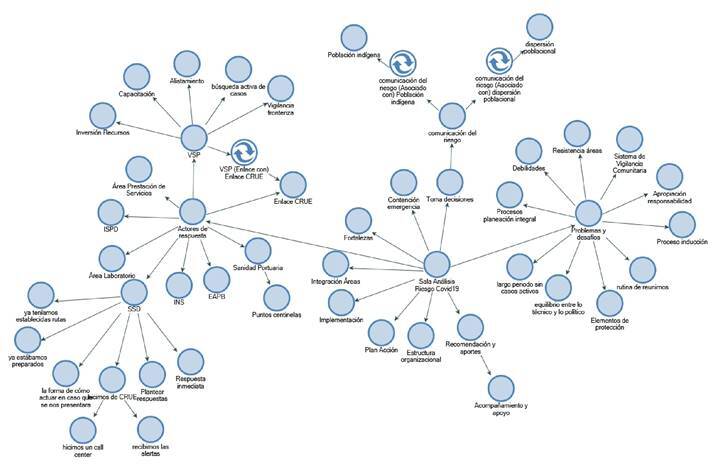
Árbol de categorías, sala de análisis del riesgo

En sus relatos, los participantes expusieron la necesidad de comunicar sobre la COVID-19, utilizando diferentes estrategias para las poblaciones indígenas y para la población rural dispersa (figura 2).

Fuente: entrevistas a participantes en las SAR (Amazonas, Guainía, Vichada y Putumayo)

*Si tuviéramos unos canales de comunicación para atender esa alerta y los demás recursos tanto urbanos como logísticos para poder hacer acciones me voy para las comunidades inmediatamente, eso sería lo ideal (ACT1001) (sic).*

*Normalmente nunca participaba comunicación del riesgo en una reunión previa si no que ya se articulaba uno con ellos cuando se quería emitir un mensaje o hacer una publicación o cuando ya se estaban haciendo acciones. Incluir a una persona en comunicación del riesgo ha sido muy importante y más por el tipo de población que nosotros tenemos, donde en un 60% es población indígena y debe ser una comunicación muy precisa y de muy fácil entendimiento (ACT1003) (sic).*

*Nosotros siempre hemos conocido las dificultades del departamento, la dispersión poblacional, la dificultad en la comunicación, pero como llegamos con lo que nosotros queremos a las comunidades para que no haya tanta mortalidad y que cada uno de su opinión y apoye esa me parece que ha sido el desafío que hemos tenido (ACT1004) (sic).*

## Discusión

Como medida inicial para enfrentar la pandemia, el Instituto Nacional de Salud adaptó el sistema de vigilancia en salud pública para incorporar la vigilancia de la COVID-19, y estableció acciones inmediatas enfocadas en la gestión del riesgo a nivel nacional y local. Como resultado de esta adaptación, se pudo verificar la implementación satisfactoria y el mantenimiento de las salas de análisis del riesgo, mediante la articulación de las diferentes áreas de cada entidad territorial fronteriza analizada. Las acciones llevadas a cabo permitieron enfocar el análisis en la gestión del riesgo de COVID-19 como una estrategia de reducción de la morbilidad y la mortalidad en el marco de eventos de alerta internacional ^27^.

Este proceso demostró cómo los procedimientos establecidos en el sistema de gestión del riesgo en Colombia desde antes de la pandemia ^4^ permitieron actuar a partir de una estructura ya establecida, lo cual facilitó la identificación, el análisis y la acción de control de la COVID-19 de manera organizada, eficiente y estandarizada desde el momento en que la OMS emitió la declaración de alerta ^28^.

Los resultados obtenidos en Colombia permiten concluir que el conocimiento generado podría fortalecer el sistema de gestión del riesgo y el sistema de vigilancia frente a futuras pandemias o situaciones similares, tal como ha ocurrido en otros países a partir de experiencias anteriores ^29^^,^^30^. Las estrategias de preparación y respuesta a la pandemia de COVID-19 se nutrieron de las ya establecidas para la pandemia de influenza A/H_1_N_1_^27^ y la epidemia del coronavirus del síndrome respiratorio de Oriente Medio (MERS-CoV) ^30^, así como de otras recientes, con el elemento común del establecimiento de un sistema de respuesta planificado bajo el esquema de evaluación del riesgo y toma de decisiones basadas en la gestión de incidentes, la vigilancia epidemiológica, la mitigación del riesgo, los protocolos de atención y tratamiento, el diagnóstico oportuno y la comunicación del riesgo ^31^.

Los departamentos de frontera analizados se caracterizan por un complejo panorama sociocultural, el difícil acceso geográfico ^24^, su gran vulnerabilidad ante la presencia de COVID-19 y la importante movilidad migratoria ^32^. No obstante estas dificultades, lograron establecer un diagnóstico inicial, planificar oportunamente acciones de vigilancia, y gestionar el recurso humano y los insumos. Parte del éxito del establecimiento de las salas de análisis del riesgo y sus componentes en los departamentos analizados, correspondería al tiempo trascurrido entre el inicio de la pandemia en marzo ^33^ y la presentación de los primeros casos (entre abril y mayo) ^34^, el cual permitió organizar la respuesta antes de la llegada del virus a cada departamento. Además, fue fundamental el apoyo oportuno del Instituto Nacional de Salud mediante los gestores que orientaron a las entidades en la respuesta.

Una vez establecidas las salas de análisis del riesgo, su continuidad permitió que cada territorio manejara su información en tiempo real y que implementara estrategias de vigilancia innovadoras, destacándose el seguimiento y la verificación de alertas y rumores, la búsqueda activa comunitaria y el tamizaje de la población vulnerable, principalmente la indígena y la de migrantes, o la que se moviliza permanentemente en las fronteras por comercio, doble nacionalidad o en búsqueda de atención médica.

La expansión y capacitación de equipos de respuesta inmediata para investigaciones epidemiológicas de campo y seguimiento de casos y contactos estrechos, permitió adoptar acciones de vigilancia y control para prevenir la propagación de la infección en las comunidades indígenas dada su vulnerabilidad. La articulación con las asociaciones indígenas fue clave para focalizar las acciones, ya que, por la autonomía del manejo de su territorio, muchas comunidades no permitieron el ingreso.

La percepción del recurso humano responsable de los procesos en las entidades territoriales sobre las salas de análisis del riesgo es que estas fortalecieron la respuesta y sirvieron de eje a un sistema integrado y organizado de las actividades de mitigación, contención y vigilancia de la pandemia. Sin embargo, este proceso integrado enfrentó dificultades ante la resistencia de áreas estratégicas frente a la articulación y planeación de procesos. En un estudio en Corea sobre la estructuración de la respuesta a la COVID-19, se demostró la importancia de la intervención y la orientación de las entidades gubernamentales del nivel nacional para establecer procesos y liderazgos a nivel local y, así, establecer metodologías sistemáticas de respuesta ante una situación desconocida. Constituye un desafío el que los actores comprendan la magnitud del desastre, y acepten la complejidad de la realidad y la necesidad de generar procesos de cooperación encaminados a un fin común ^35^.

Las dificultades en la articulación inicial de las áreas de salud en los territorios evaluados en Colombia, pueden ser una respuesta ante la incertidumbre administrativa y el miedo frente a un evento desconocido, no solo localmente sino también a nivel mundial. Su posterior articulación y planeación de procesos pueden responder, entonces, a un mayor conocimiento y apoyo nacional en la preparación de la respuesta, lograda con la ayuda de los gestores del Instituto Nacional de Salud.

En los territorios analizados, la sala de análisis de riesgo, apoyada en su implementación y mantenimiento por un gestor representante de una entidad nacional, generó capacidades para responder a la pandemia de COVID-19 de forma oportuna e inmediata, estableciendo procesos que facilitaron su vigilancia epidemiológica y un espacio de discusión para la toma de decisiones efectivas orientadas a la contención y posterior mitigación del riesgo en cada entidad territorial, fortaleciendo así los pasos fronterizos en Colombia.

La operación del sistema de vigilancia comunitaria y la comunicación del riesgo, específicamente en la población indígena, son factores que deben mejorarse en los territorios. Estos procesos requieren de una evaluación a fondo que logre generar un diagnóstico y una estrategia de trabajo que contemplen las dificultades de acceso a las comunidades, la inexistencia de medios de comunicación en gran parte de las localidades rurales y la gran variedad étnica y cultural de los territorios.

Se establecieron estrategias efectivas que pueden aportar a la resolución de los problemas comunitarios en la población indígena o rural dispersa durante la pandemia. En México, cuyas poblaciones indígenas son más vulnerables ante la COVID-19 por diversos factores sociales, ambientales y de salud, se ha detectado la necesidad de establecer esquemas de base comunitaria que permitan llegar a estas poblaciones para responder a sus necesidades ^36^. En el marco de los factores sociales determinantes de la salud, los sistemas de salud pueden mitigar los impactos de la pandemia mediante acciones de comunicación eficaz, incluida la comunicación del riesgo, que aumenten la confianza y la credibilidad en las autoridades y en las fuentes de información. Asimismo, la comunicación del riesgo debe llegar principalmente a la población pobre y vulnerable, con especificidades locales en términos de idioma y cultura ^37^.

La pandemia deja un mensaje claro acerca de la importancia del componente de comunicación y las medidas de prevención; es por ello que, desde las salas de análisis de riesgo que se implementaron en cada entidad territorial, se involucraron diversos sectores para establecer planes de comunicación orientados a la prevención y el control del nuevo coronavirus. Este hallazgo es similar al de un estudio en Cuba, donde se evidenció la importancia de establecer planes de comunicación de crisis y programas integrales de comunicación social para orientar a la población en la prevención y control de la COVID-19 ^38^.

Las salas de análisis del riesgo constituyen un esfuerzo conjunto del nivel nacional y local que promueve la participación articulada de las partes involucradas, para analizar información y optimizar la respuesta organizada durante la pandemia. Determinar las debilidades y fortalezas del proceso de su implementación y mantenimiento, es fundamental para conocer los desafíos y mejorar los planes estratégicos de gestión del riesgo en futuras emergencias de salud pública.

Se recomienda a los departamentos mantener las salas de análisis del riesgo después de la pandemia, con el fin de hacer gestión del riesgo en las situaciones de interés en salud pública.
